# Beam energy metrics for the acceptance and quality assurance of Halcyon linear accelerator

**DOI:** 10.1002/acm2.13281

**Published:** 2021-05-27

**Authors:** Song Gao, Mikhail A. Chetvertkov, Bin Cai, Abhishek Dwivedi, Dimitris Mihailidis, Xenia Ray, Tucker Netherton, Laurence E. Court, William E. Simon, Peter A. Balter

**Affiliations:** ^1^ Department of Radiation Physics The University of Texas MD Anderson Cancer Center Houston TX USA; ^2^ GenesisCareUS Madison Heights MI USA; ^3^ Radiation Oncology Washington University School of Medicine St. Louis MO USA; ^4^ Radiation Oncology Rutgers CINJ/Robert Wood Johnson University Hospital New Brunswick NJ USA; ^5^ Radiation Oncology University of Pennsylvania Philadelphia PA USA; ^6^ Radiation Medicine and Applied Science University of California San Diego Moores Cancer Center La Jolla CA USA; ^7^ Sun Nuclear Corporation Melbourne FL USA

**Keywords:** acceptance and QA, Halcyon linear accelerator, ionization chamber array, monitoring energy

## Abstract

**Purpose:**

Establish and compare two metrics for monitoring beam energy changes in the Halcyon platform and evaluate the accuracy of these metrics across multiple Halcyon linacs.

**Method:**

The first energy metric is derived from the diagonal normalized flatness (F_DN_), which is defined as the ratio of the average measurements at a fixed off‐axis equal distance along the open profiles in two diagonals to the measurement at the central axis with an ionization chamber array (ICA). The second energy metric comes from the area ratio (AR) of the quad wedge (QW) profiles measured with the QW on the top of the ICA. Beam energy is changed by adjusting the magnetron current in a non‐clinical Halcyon. With D_10cm_ measured in water at each beam energy, the relationships between F_DN_ or AR energy metrics to D_10cm_ in water is established with linear regression across six energy settings. The coefficients from these regressions allow D_10cm_(F_DN_) calculation from F_DN_ using open profiles and D_10cm_(QW) calculation from AR using QW profiles.

**Results:**

Five Halcyon linacs from five institutions were used to evaluate the accuracy of the D_10cm_(F_DN_) and the D_10cm_(QW) energy metrics by comparing to the D_10cm_ values computed from the treatment planning system (TPS) and D_10cm_ measured in water. For the five linacs, the D_10cm_(F_DN_) reported by the ICA based on F_DN_ from open profiles agreed with that calculated by TPS within –0.29 ± 0.23% and 0.61% maximum discrepancy; the D_10cm_(QW) reported by the QW profiles agreed with that calculated by TPS within –0.82 ± 1.27% and –2.43% maximum discrepancy.

**Conclusion:**

The F_DN_‐based energy metric D_10cm_(F_DN_) can be used for acceptance testing of beam energy, and also for the verification of energy in periodic quality assurance (QA) processes.

## 1 Introduction

The Halcyon (Varian Medical Systems, Palo Alto CA) is a single‐energy 6‐MV flattening filter free photon beam machine, and the universal beam model in the Eclipse (Varian Medical Systems, Palo Alto CA) treatment planning system (TPS) is predefined for all Halcyon linear accelerators (linacs).

A recent study demonstrated that the Halcyon platform can be validated with an ionization chamber array (ICA) and a 1D water scanner (1DS) without the need for a 3D water scanning system.^1^ The commissioning verification was based on the AAPM Medical Physics Practice Guideline for Commissioning and QA of External Beam Planning Systems (MPPG5.a).^2^ The diagonal normalized flatness (F_DN_), which is calculated from open beam profiles measured with an ICA, was verified as a metric for monitoring beam energy and was more sensitive and reproducible than the traditional percent depth dose (PDD) energy metric.^3^, ^4^ Another method for determining photon beam energy uses a quad wedge (QW) which consists of two pairs of copper wedge‐shaped attenuators along each of the diagonal detector axes of the ICA, with the wedge pairs being symmetrically opposed and the thin sections toward the array center.^5^ The energy metric from the QW is the area ratio (AR), which is defined as the cumulative of measurements from a span of detectors in the presence of wedges and normalized to the cumulative of measurements from a similar detector set on the X and Y axes (open field profiles).^5^

The purpose of this study was to establish a legitimate ICA method for D_10cm_ verification for the Halcyon linac for both commissioning and periodic quality assurance, without the cumbersome 1DS water tank.

### 1.1 Summary of terminology

F_DN_: the diagonal normalized flatness, the ratio of the average measurements at a fixed off‐axis equal distance along the profiles in two diagonals to the measurement at the central axis.

QW: a quad wedge plate consists of two pairs of copper wedge‐shaped attenuators along each of the diagonal detector axes.

AR: the area ratio (AR) which is defined as the sum of ICA measurements from a span of detectors under a quad wedge (QW) and normalized to the sum of measurements from a similar detector set on the X and Y axes (open field profiles).

D_10cm_(water): Percent depth dose (PDD) at depth 10 cm, measured in water with 1D scanner (1DS). For simplicity, we use D_10_ denote D_10cm_ hereafter in the text.

D_10_(TPS): PDD at 10 cm computed from the treatment planning system (TPS).

D_10_(F_DN_): PDD at 10 cm computed from F_DN_, which is calculated from open beam profiles measured with an ICA.

D_10_(QW): PDD at 10 cm computed from the AR with QW profiles measured using an ICA.

## 2 MATERIALS AND METHODS

Both F_DN_‐based and AR‐based energy metrics require calibration against a known energy metric that is chosen to be the percent depth dose (PDD) at a depth of 10 cm, D_10_(water), measured in water at 90 cm source to surface distance (SSD). This setup matches that the vendor provided reference data which were specified at 90 cm SSD due to the geometry limit of the machine bore. To establish the relationships between F_DN_ and AR with D_10_ in water, beam energy is changed by adjusting the magnetron current in a non‐clinical Halcyon corresponding to a change in beam energy from −10% to +5% off from its nominal value (Note that +10% produced an unstable beam). At each magnetron current setting, ICA measurements are acquired with an IC Profiler (Sun Nuclear, Melbourne FL) in open beam profiles and profiles with quad wedge (QW) (Sun Nuclear, Melbourne FL) for a maximum field size of 28 × 28 cm^2^. In addition, PDDs were scanned in the 1D Scanner (1DS) (Sun Nuclear, Melbourne FL), providing a relationship between D_10_ and F_DN_ and AR, both calculated from the ICA measurements. Once these relationships are established with linear data fitting, D_10_ values can be calculated from D_10_(F_DN_) or D_10_(QW), enabling future D_10_ measurements with only the ICA, without a water tank.

To evaluate the trueness of F_DN_‐based and AR‐based energy metrics in predicting D_10_ values, open and QW beam profiles were measured with ICAs in five Halcyon linacs from five institutions under the same setup condition. D_10_(F_DN_) and D_10_(QW) were calculated from the fitted functions from the non‐clinical Halcyon linac and then compared with the D_10_(water) and D_10_(TPS) specific to each of the five clinical Halcyon linacs.

### 2.1 Measurement setup and procedure

The Halcyon is an inline magnetron linear accelerator with no bending magnet, the beam energy can be changed by adjusting the magnetron current. Without a direct means of determining how changes in magnetron current (MI) would change energy, previously determined relationships were used between changes in diagonal normalized flatness F_DN_ and changes in energy on a TrueBeam 6‐MV FFF beam.^3^ Each energy metric was measured at the nominal beam energy and at five intentionally created energy changes off –10.0%, –5.0%, –2.5%, +2.5%, and +5.0% from the nominal beam energy value (0%). After beam tuning to achieve stable dose rates and symmetric beam profiles for each MI setting, the beam parameters were saved to a file and later loaded for each tuned beam, which provided reproducible measurement setups.

**Fig. 1:**
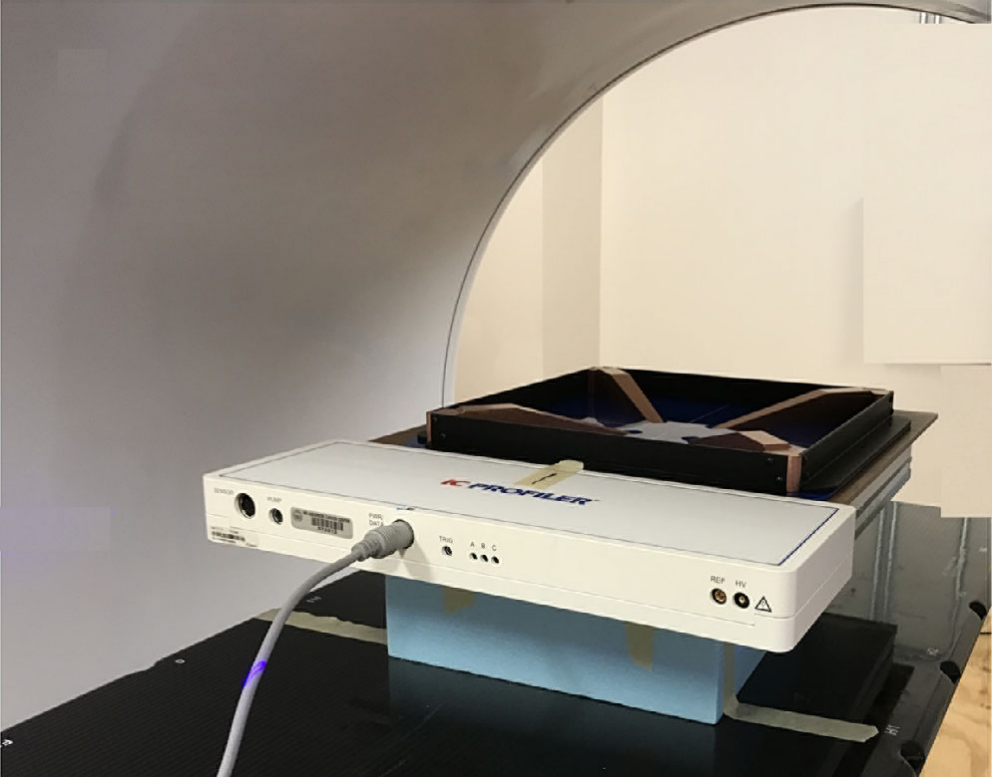
The IC array measurement setup positions with solid water buildup and QW plate.

Because the Halcyon does not have light field and radiation isocenter lasers, the ICA was set up in the following three steps^1^: (1) initial alignment with the outside bore lasers (virtual isocenter); (2) image guidance for final alignment with two orthogonal MV images; (3) acquisition of test profiles to verify the beam center and for fine‐tuning centering. The final positions for measurements with solid water buildup and the QW plate are illustrated in Fig. 1.

At each magnetron current setting, beam profiles were measured with an ICA with and without the QW. The ICA was then removed and PDDs were scanned with a 1DS with a 90‐cm SSD for each energy.

### 2.2 Percent depth dose at changes in beam energy

PDD scans were obtained in a 1DS with SNC125c (Sun Nuclear, Melbourne FL) waterproof active volume of 0.125 cm^3^ ion chambers and a PC electrometer (Sun Nuclear, Melbourne FL) by SNC dosimetry software. PDDs were scanned at six MI settings corresponding to the nominal beam energy and five intentional energy changes from the nominal energy. All PDD measurements were obtained at 90 cm SSD with 10 × 10 cm^2^ field size at scanning depths ranging from 0 to 30 cm. The data were smoothed and renormalized to 100% by the values at d_max_ for each energy.

### 2.3 ICA calibration and profile measurement

The detectors in the ICA were calibrated at the nominal beam energy on Halcyon linac at a detector depth of d_max_ (1.4 cm = 0.9 cm inherent + 0.5 cm solid water), at an extended 110‐cm SSD and 28 × 28 cm^2^ field. The divergent field from the 100‐cm SSD produced a 30.8 × 30.8 cm^2^ field, which ensures that all detectors used in later measurements at 90 cm SSD were within the calibration field. The accuracy of the array calibration was evaluated according to previously proposed procedures,^6^ and the calibration uncertainties were <0.5% for all detectors in the measurement field.

Beam profiles were measured for the maximum field size, with the ICA and at depth d_max_, with 90 cm SSD. Profiles were measured on four axes, in‐plane, cross‐plane, and diagonals at six MI settings corresponding to the nominal beam energy and five intentionally created energy changes from the nominal energy, using the single calibration measurement at the nominal beam energy.

### 2.4 Beam energy metrics

The percent depth dose at 10 cm depth, D_10_(water), from the PDD scanned in the water tank at 90 cm SSD with 10 × 10 cm^2^ field size provided the standard energy metric.

#### 2.4.1 Diagonal normalized flatness energy metric

In beam profiles acquired with ICA, the diagonal normalized flatness is functionally related to beam energy and can be used as an energy metric.^3^, ^4^ The F_DN_ values at different off‐axis distances can be obtained from the open profiles through the IC PROFILER software. The off‐axis points in the F_DN_ are the points at the off‐axis distances of approximately 90% and 60% of the maximum beam intensity, which are ±5.7 cm and ±15.6 cm, respectively, from the array center and located in a stable region of the profile. The relationship between D_10_(water) and F_DN_ from ICA measurement is calibrated by acquiring profiles at the six beam energies. A linear fit is determined between F_DN_ and D_10_(water) that can then be used to measure D_10_ from F_DN_ obtained from the ICA profile:

(1) $$D_{10}\left(F_{\text{DN}}\right)=n\cdot F_{\text{DN}}+a,$$

where *n* and *a* are the slope and intercept values. Once this relationship is established, the D_10_(F_DN_) reports the value of D_10_ at 90 cm SSD with 10 × 10 cm^2^ field size.

#### 2.4.2 Quad wedge profile energy metric

Another method for determining photon beam energy is from the beam profiles acquired using ICA with a quad wedge (QW) on top of the ICA surface. The two wedge pairs in the QW plate are symmetrically opposed to the thin sections toward the array center (see Fig. 1). In beam profiles acquired with ICA and QW combination, the AR under the wedges is related to the beam energy, and thus the AR can be used as an energy metric.

The AR from the QW profiles is the sum of measurement data under the wedges (along diagonal axes) normalized to the sum of measurement data from a similar detector set on the X and Y axes (open field profiles)^5^:

(2) $$\text{AR}=\frac{{\text{PD}}_{\text{Area}}+{\text{ND}}_{\text{Area}}}{X_{\text{Area}}+Y_{\text{Area}}},$$

where PD_Area_, ND_Area_, X_Area_, and Y_Area_ represent the sum of corrected counts from the applicable detectors of the measured profiles for positive diagonal (PD), negative diagonal (ND), X, and Y axes, respectively.

The relationship between the measured AR and D_10_(water) is established by determining the AR from the QW profiles at six beam energies with known D_10_(water). A linear fit is determined between AR and D_10_(water), which can then be used to determine D_10_(AR) from the measured profile with ICA/QW on a beam from the Halcyon linac:

(3) $$D_{10}\left(\text{AR}\right)=m\cdot \text{AR}+b,$$

where *m* and *b* are the slope and intercept values and AR is the area ratio. Similar to D_10_(F_DN_), D_10_(AR) also reports the value of D_10_ at 90 cm SSD with 10 × 10 cm^2^ field size.

#### 2.4.3 Evaluation of beam energy metrics

Once the D_10_(F_DN_) and D_10_(QW) metrics were established in the non‐clinical Halcyon, meaning that the linear coefficients [eqs. (1) and (3), respectively] were derived from the calibrations, the IC Profiler software could report D10(F_DN_) or D10(QW) from the measured open or QW profile, respectively. Five Halcyon linacs from five institutions were selected to study beam energy results by the methods D_10_(water), D_10_(TPS), D_10_(F_DN_), and D_10_(QW). The D_10_(water) and D_10_(TPS) metrics were obtained on each institution’s clinical Halcyon, as was used in the non‐clinical Halcyon, and the D_10_(TPS) was calculated with the institution’s clinical Eclipse TPS.

D_10_(F_DN_) and D_10_(QW) were calculated from their respective metrics in the five linacs at the institutions’ nominal clinical beam energy. D_10_(F_DN_) and D_10_(QW) were then compared with the measured PDD value D_10_(water) and the calculated D_10_(TPS). Their mean differences and corresponding standard deviations are presented below in the Results.

## 3 RESULTS

### 3.1 Changes in percent depth dose

**Fig. 2:**
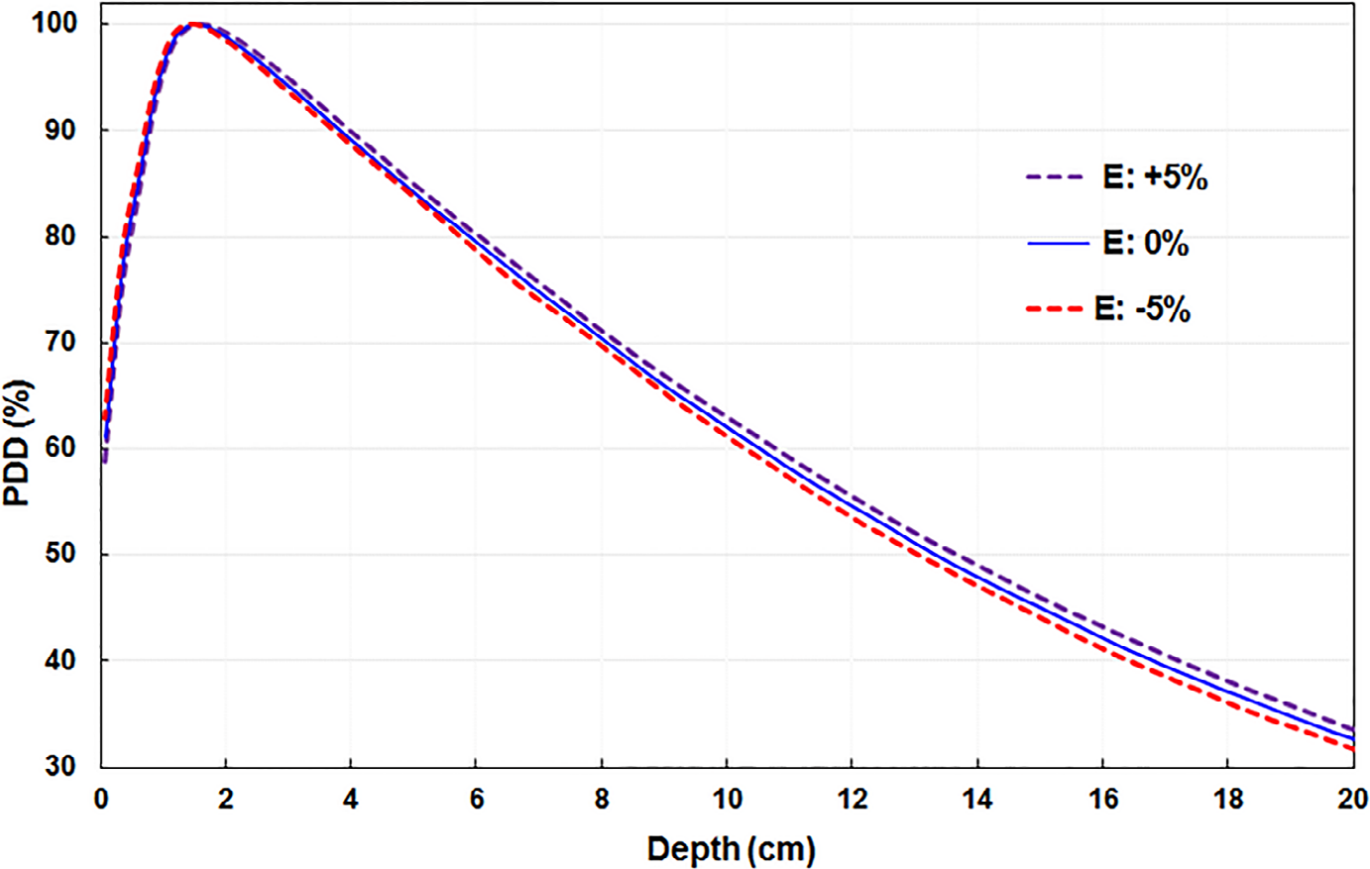
The percent depth dose (PDD) scanned using the 1D water tank (shown for up to depth 20 cm only) with a source‐to‐surface distance (SSD) of 90 cm for field of 10 × 10 cm^2^ for nominal beam energy (E: 0%), and energy changes +5% (E: +5%) and −5% (E: −5%) from nominal energy.

The PDD data were measured for 10 × 10 cm^2^ field size with 90 cm SSD by the 1D water scanning system at the nominal clinical beam energy and at five energy changes from nominal (0%): –10.0%, –5.0%, –2.5%, +2.5%, and +5.0%. The PDD curves were normalized to d_max_ depth for each energy, and an example of the effect of changes in beam energy on the changes in PDD curves is presented in Fig. 2. The D_10_ values of these six beam energies can be obtained from the PDD curves.

### 3.2 Changes in the profile

**Fig. 3:**
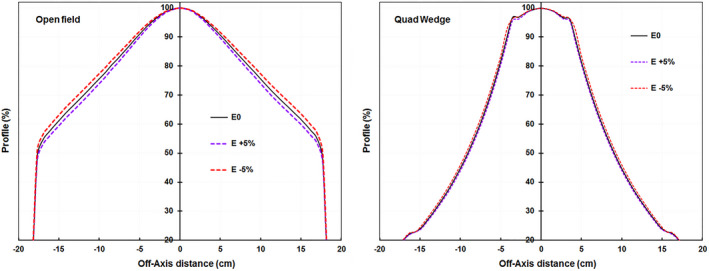
Diagonal profiles measured with an ionization chamber profiler (ICA) with open field (left) or with quad wedge atop the ICA (right) for nominal beam energy (E: 0%), and energy changes +5% (E: +5%) and −5% (E: −5%) from nominal energy. The source‐to‐surface (SSD) is 90 cm, depth d_max_, and the field size is 28 × 28 cm^2^.

Beam profiles were measured at d_max_, 90 cm SSD with a 28 × 28 cm^2^ field size with and without QW, at the six beam energies (nominal clinical energy and five adjusted energy beams). The profiles were normalized to the corresponding central axis value for each beam energy, revealing the shape change in the profiles with the variation of the beam energy (Fig. 3).

### 3.3 Beam energy metrics

**Fig. 4:**
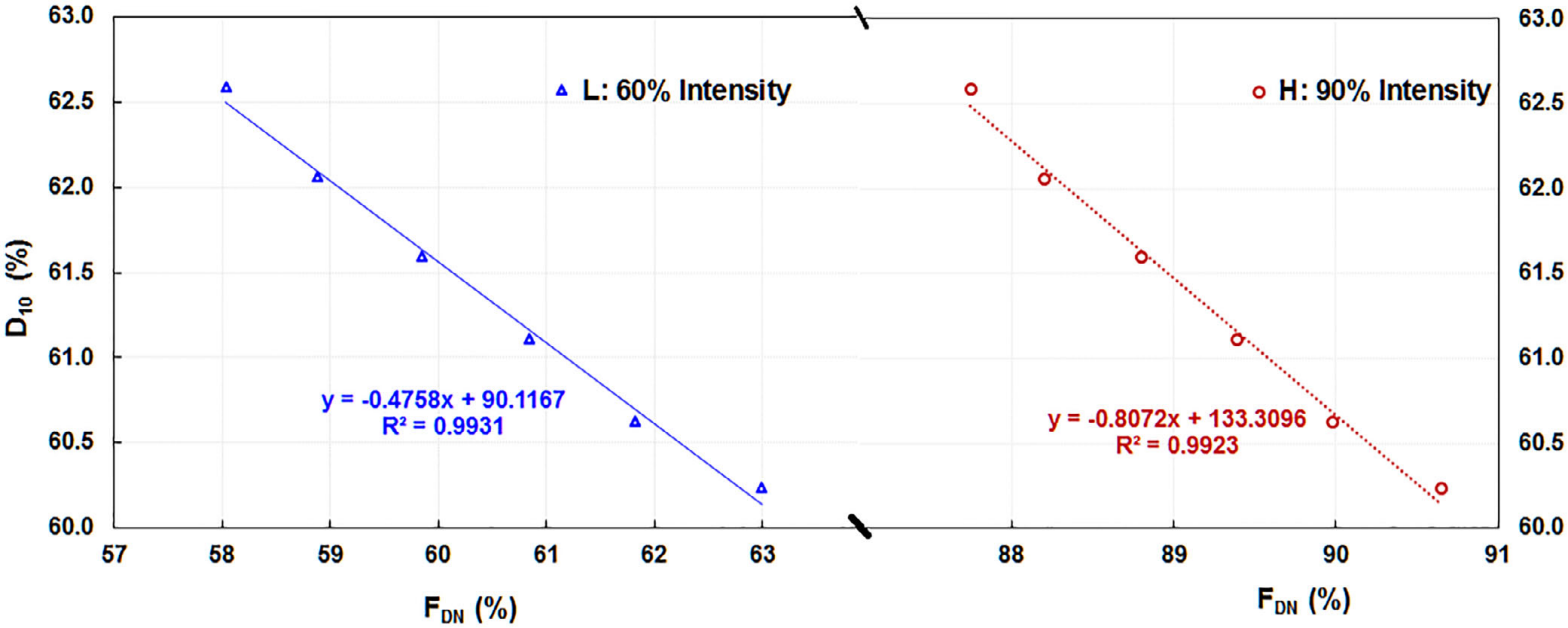
The linear relationship between the percent depth dose at 10 cm depth D_10_ scanned with the 1D water scanner (1DS) and the diagonal normalized flatness (F_DN_) measured from the beam profile with the ionization chamber array (ICA) for beam energies off the nominal beam energy by –10.0%, –5.0%, –2.5%, +2.5% and +5.0%. The off‐axis distances are approximately 60% (L) and 90% (H) of maximum beam intensity.

From the F_DN_ values at different off‐axis distances and the corresponding D_10_(water) values of the six beam energies, the linear fit parameters of the relationship between D_10_(water) and F_DN_ are summarized in eqs. (4) and (5) (Fig. 4):

(4) $$D_{10}\left(F_{\text{DN}}\right)=‐0.807\cdot F_{\text{DN}}+133.3,\;F_{\text{DN}}\;\text{at}\;\pm 5.7\;\text{cm};{}^{\prime }H^{\prime \prime }$$

(5) $$D_{10}\left(F_{\text{DN}}\right)=‐0.476\cdot F_{\text{DN}}+90.1,\;F_{\text{DN}}\;\text{at}\;\pm 15.6\;\text{cm};{}^{\prime }L^{\prime \prime }$$

where H designates the high‐intensity location on the profile and L the low‐intensity location. D_10_(F_DN_) is calculated from the F_DN_ values measured with ICA by the equation above and compared with the D_10_(water) values for all testing beams (Table 1). The D_10_(F_DN_) results from locations H and L have a maximum deviation ±0.02%.

**Table 1:** Comparison of the percent depth dose at a depth of 10 cm (D_10_), scanned with a 1D water scanning system, and the D_10_ values determined from the diagonal normalized flatness (F_DN_) at two off‐axis distances, 90% (H) and 60% (L) of maximum beam intensity, for five beam energies off the nominal energy (by –10.0%, –5.0%, –2.5%, 0%, +2.5% and +5.0%). Also shown are average values and differences (δ) for the difference in D_10_ between F_DN_ (average) and 1DS; the D_10_ determined from quad‐wedge profile (QW); and differences in D_10_ between QW and 1DS. Data from one non‐clinical Halcyon linac.

Change in energy (%)	5.0%	2.5%	0.0%	−2.5%	−5.0%	−10.0%
D_10_(water), %	62.59	62.07	61.60	61.12	60.63	60.24
D_10_(F_DN_),%						
H	62.48	62.12	61.64	61.16	60.69	60.14
L	62.51	62.10	61.64	61.17	60.70	60.14
Average	62.49	62.11	61.64	61.16	60.69	60.14
δ (H–L)	−0.02	0.02	0.00	−0.01	−0.02	0.00
δD_10_(F_DN_‐water)	−0.10	0.04	0.04	0.05	0.06	−0.10
D_10_(QW),%	62.43	62.08	61.61	61.17	60.69	60.04
δD_10_(QW‐water)	−0.16	0.02	0.01	0.05	0.06	−0.20

**Fig. 5:**
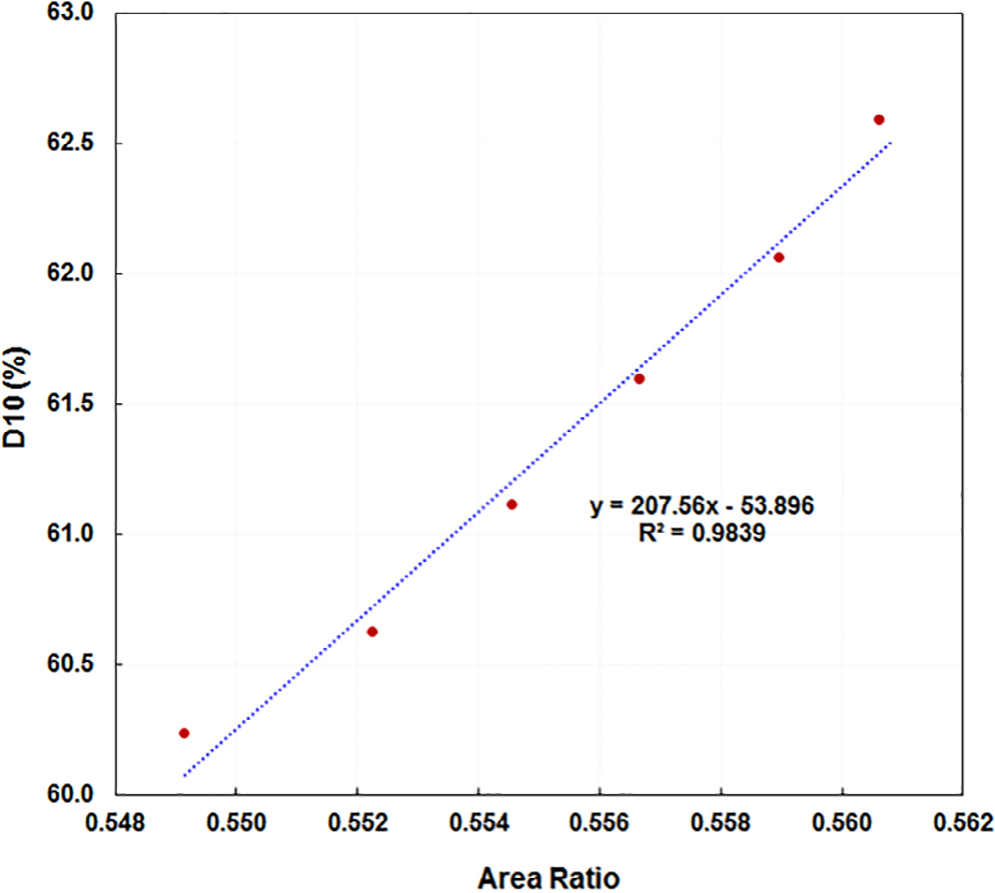
The linear relationship between the percent depth dose at 10 cm depth D_10_ scanned using the 1D water scanner (1DS) and the area ratio from the QW profile measured with the ionization chamber array (ICA) and QW plate for beam energies off the nominal beam energy by –10.0%, –5.0%, –2.5%, +2.5% and +5.0%.

From the AR values from the QW profiles and the corresponding D_10_(water) values of the six beam energies, the linear fit parameters of the relationship between D_10_(water) and AR are summarized in Eq. (6) (Fig. 5):

(6) $$D_{10}\left(\text{QW}\right)=207.66\cdot \text{AR}‐53.90.$$

With this calibration, the IC PROFILER software can report the D_10_(QW) values from the QW profiles. The D_10_(QW) values were compared with the corresponding D_10_(water) values for all six testing beams (Table 1). The differences are within 0.2%. It should be noted that the slope and intercept values in eqs. (4)–(6) are only valid for a field size of 28 × 28 cm^2^ and SSD of 90 cm for profiles measured using ICA and the D_10_(water) values used for calibration were measured at a field size of 10 × 10 cm^2^ and SSD of 90 cm in the water tank.

### 3.4 Evaluation of beam energy metrics

The measured D_10_(F_DN_) and D_10_(QW) values from five Halcyon linacs were compared with the measured D_10_(water) and calculated D_10_(TPS) values (Table 2).

**Table 2:** The percent depth dose at a depth of 10 cm (D_10_) determined from the diagonal normalized flatness (F_DN_) and from the quad‐wedge (QW) profile of five Halcyon linacs from five institutions. Also shown are the difference in D_10_ between F_DN_ and TPS (QW and TPS) and the difference in D_10_ between F_DN_ and water scans (QW and water scans), along with their averages and standard deviations (σ).

Method	Off‐axis ratio (F_DN_)			Quad Wedge (QW)		
Machine	D_10_ (F_DN_)	δD_10_ (F_DN_‐TPS)	δD_10_ (F_DN_‐water)	D_10_ (QW)	δD_10_ (QW‐TPS)	δD_10_ (QW‐water)
M1	61.75	0.33	0.15	61.74	0.32	0.14
M2	62.03	0.61	0.18	63.85	2.43	2.00
M3	61.83	0.41	0.43	63.64	2.22	2.24
M4	61.61	0.19	0.71	61.12	–0.30	0.22
M5	61.33	–0.09	–0.47	60.82	–0.60	–0.98
Average	61.71	0.29	0.22	62.24	0.82	0.22
σ	0.23	0.23	0.20	1.27	1.27	0.20

The differences of D_10_ between F_DN_ and treatment planning (TPS) calculations [D_10_(F_DN_) – D_10_(TPS)] produced an average ± standard deviation (σ) of 0.29 ± 0.23%, with a range from 0.61% to –0.09%. Likewise, the differences [D_10_(F_DN_) – D_10_(water)] in D_10_ between F_DN_ and water scans [D_10_(F_DN_) – D_10_(water)] were an average of 0.22 ± 0.20%, with a range from 0.71% to –0.47%.

Comparing D_10_ values determined from the QW profile to the TPS calculations and in‐water measurements, the difference in D_10_ between the QW measurements and TPS calculations [D_10_(QW) – D_10_(TPS)] was an average ± standard deviation of 0.82 ± 1.27%, with a range from 2.43% to –0.60%. The difference in D_10_ between QW and in‐water measurements [D_10_(QW) – D_10_(water)] was 0.22 ± 0.20%, with a range from 2.24% to –0.98%.

In summary, results from each of the five Halcyon linacs tested indicate that the D_10_ values determined by F_DN_ from the open beam profiles agree with TPS calculations and in‐water measurements within 0.8% for all linacs; and the D_10_ values determined by the QW profiles agree with TPS calculations and in‐water measurements within 1.0% for three linacs but up to 2.4% for two linacs. This discrepancy could come from the differences in collimator scatter and/or electron contamination in the low buildup region of the detectors, especially detectors in the X and Y axes.

## 4 DISCUSSION

The Halcyon linac and the associated Eclipse TPS model are provided as an integrated system with no user‐specific beam modeling capability, and thus the data collection at the time of acceptance testing and subsequent commissioning can be restricted to the need to verify rather than to create the TPS model.^1^, ^7^ A 2D ionization chamber array (ICA) can be used to measure changes in the energy of photon beams with a higher sensitivity than with PDD measurements.^3^, ^4^ This work demonstrated that with proper calibration, the diagonal F_DN_ measured with the ICA can be used to predict D_10_ that matched the TPS calculation and PDD water tank data. The measured D_10_(QW) values also agreed reasonably well with the TPS calculation and the in‐water measurement.

To examine the uncertainty of D_10_ values from linear regression, we excluded the minimum and the maximum points which are not fit the linear regression lines very well in Figs. 4 and 5. We obtained better coefficient of determination R^2^ in the linear fit, D_10_(F_DN_) = –0.4885 F_DN_ + 90.8326, R^2^ = 0.9999) and D_10_(F_DN_) = –0.8106 F_DN_ +133.5704, R^2^ = 0.9998 corresponding to L: 60% intensity and H 90% intensity in Fig. 4, respectively. We calculated the D_10_(F_DN_) values of all five linacs using these new linear fit equations. The difference of D_10_(F_DN_) values between the previous linear fit and the new linear fit (minimum and the maximum points excluded) is −0.04 ± 0.01% with a maximum of −0.05%. Similarly, for quad‐wedge‐based profile metric, we obtained D_10_(QW) = 214.5833AR – 57.8710, R^2^ = 0.9994 after excluded the minimum and the maximum points in the linear fit from the QW profiles (corresponding to Fig. 5). We calculated the D_10_(QW) values of all five linacs using this new linear fit. The difference of D_10_(QW) values between the previous linear fit and the new linear fit (minimum and the maximum points excluded) is −0.03 ± 0.07% with a maximum of 0.13%.

For beam energy verification, although the diagonal F_DN_ method and QW profile method gave similar results for the non‐clinical linac used for calibrations, the diagonal F_DN_ method showed better agreement than the QW method to TPS and in‐water data for all other tested linacs. The QW method integrates selected detectors along the portion of diagonal axes under the QW and divides that by a selected portion of detectors along the X and Y axes, resulting in an “Area Ratio.” The detectors along the portion of diagonal axes and along the X and Y axes have different collimator scatter and/or electron contamination in the low‐buildup region because of differences in the thickness of wedge material or no wedge, which could introduce measurement uncertainty. The F_DN_ method is preferred because it does not require an additional device, and measurements and array calibration are done under the same buildup condition. In the quad‐wedge profile method, the ICA array calibration was performed without a quad‐wedge plate on top of the ICA, but the quad‐wedge plate was in the beam path during the measurement. The copper wedge attenuates beam intensity as well as generating scatter radiation, which could increase measurement uncertainty. According to TG 142 recommendations,^8^ X‐ray beam quality D_10_ should be ±1% from baseline in annual QA checks. The D_10_ from F_DN_ metric has variation within this tolerance level, and the D_10_ from QW metric variation is outside this window. For simplicity and clean data acquisition, we recommend using the diagonal F_DN_ method for energy verification.

## 5 Conclusions

The diagonal normalized flatness (F_DN_) from the ICA beam profile energy metric and QW profile‐based energy metric were established for Halcyon. The uncertainty analyses indicated the maximum linear fit induced error in D_10_(F_DN_) is less than 0.06%, and in D_10_(QW) is less than 0.14% which is not significant. The results of applying these independent energy metrics to five Halcyon linacs from five institutions demonstrated that the F_DN_‐based energy metric D_10_(F_DN_) is better matched to the D_10_(TPS) and D_10_(PDD) values than those measured with the quad‐wedge profile‐based energy metric D_10_(QW). The energy metric D_10_(F_DN_) can be used for acceptance of beam energy, for verification of energy in periodic QA testing, and also greatly speeds up the acceptance and QA processes.

## CONFLICTS OF INTEREST

The authors have no conflict of interest to disclose.

## AUTHOR CONTRIBUTIONS

Peter A. Balter, Song Gao, William E. Simon, Laurence E. Court: designed the study. Song Gao, Mikhail A. Chetvertkov, Bin Cai, Abhishek Dwivedi, Dimitris Mihailidis, Xenia Ray, Tucker Netherton: collected beam data and analyzed results. Song Gao, William E. Simon, Dimitris Mihailidis: wrote this manuscript. All contributing authors: edited this manuscript.

## Data Availability

The data that support the findings of this study are available from the corresponding author upon reasonable request.
